# Fungal Biotechnology and Applications

**DOI:** 10.3390/jof9090871

**Published:** 2023-08-24

**Authors:** Baojun Xu

**Affiliations:** Food Science and Technology Program, BNU-HKBU United International College, Zhuhai 519087, China; baojunxu@uic.edu.cn; Tel.: +86-756-3620636

The demand for fossil fuels for industry, agriculture, transportation, and private sectors is sharply increasing globally. An insufficient fuel supply can be alleviated using alternative fuels. In the future bio-based society, a wide range of challenges will have to be solved by new biological solutions. For example, bioethanol is a liquid biofuel produced from biomass via the microbial fermentation of various biomass substrates, such as lignocelluloses [1]. Millions of tons of agro-industrial waste byproducts are generated annually from agricultural practices on lignocellulose biomasses worldwide. Straws of crops are paradigms of agro-industrial waste byproducts containing lignocelluloses [2,3]. Among the core technologies for these transformations are the fungal production of proteins, metabolites and chemicals and biological processing. However, there is still a significant gap in our knowledge about the biological and chemical properties of fungal. Thus, this Special Issue discusses the latest knowledge and critical thinking on fungal biotechnology and its applications. This Special Issue provides an enabling platform for innovative academics, engineers and industrial experts in the field of fungal biotechnology and food science to exchange new ideas and present research results. This Special Issue includes nine original research papers and four reviews on recent findings in the field.

The thermotolerant yeast *Saccharomyces cerevisiae* has received considerable interest for industrial ethanol production. In a study conducted by Boonchuay et al. [1], an effective thermotolerant yeast strain S. cerevisiae TC-5 was selected for bioethanol production under elevated temperatures via simultaneous saccharification and fermentation SSF and the use of cellulose-rich corncob (CRC) residue. The maximum ethanol concentration and ethanol productivity values were 31.96 g/L and 0.222 g/L/h, respectively. In another study [4], a cDNA library was constructed from the mRNA of a methylotrophic yeast, *Ogataea polymorpha*, and then introduced into S. cerevisiae using horizontal gene transfer technology, a process through which an organism acquires genes from other organisms. The results confirmed that heat tolerance in yeast is a complex phenotype dependent on multiple quantitative loci. Artificial random gene transfer can emerge as a valuable tool in yeast bioengineering to investigate the background of complex phenotypes, such as heat tolerance.

As β-glucosidases represent the major bottleneck for the industrial degradation of plant biomass, great efforts are being devoted to developing efficient and inexpensive ways to produce them. In their study, Méndez-Líter et al. [5] utilized raw glycerol as an appropriate carbon source to produce efficient enzyme cocktails with high β-glucosidase levels using the fungus *Talaromyces amestolkiae*. The crude enzyme was successfully used to supplement a basal commercial cellulolytic cocktail for the saccharification of pretreated wheat straw, corroborating that even hardly exploitable industrial wastes, such as glycerol, can be used as secondary raw materials to produce valuable enzymatic preparations in a framework of the circular economy.

A research work [3] highlighted the valorization of the bulky recalcitrant lignocellulose byproduct of wheat straw (WS) for the enhanced production of value-added xylanase by the locally sourced novel *Penicillium chrysogenum* strain A3 DSM105774. The optimized production of xylanase via a submerged state of fermentation of WS was achieved using a three-step statistical and sequential approach.

Solid state fermentation (SsF) is recognized as a suitable process to produce enzymes using organic residues as substrates. However, only a few studies have integrated an evaluation of the feasibility of applying enzymes produced by SsF into subsequent hydrolyses followed by the production of target compounds, e.g., lactic acid (LA), through submerged-liquid fermentations (SmF). In another study [6], wheat bran (WB) was used as the substrate to produce enzymes via SsF by *Aspergillus awamori* DSM No. 63272. These enzymes were then used for the hydrolysis of the organic fraction of municipal solid waste (OFMSW). Subsequently, hydrolysates were fermented in SmF by *Bacillus coagulans* A166, increasing the LA concentration by 15.59 g/L. The data reported in this study provide an example of how SsF and SmF technologies can be combined for the valorization of WB and OFMSW.

The potential of rose flowers and lavender straw waste biomass was studied [7] as feed for lignocellulose substrates for the cultivation of the newly isolated *Ganoderma resinaceum* GA1M in Bulgaria, with the objective of obtaining mycelium-based bio-composites.

The bicarbonate (HCO_3_^−^) transporter family, including the anion exchanger (AE) group, is involved in multiple physiological processes through regulating acid–base homeostasis. HCO_3_^−^ transporters have been extensively studied in mammals, but fungal homologues of AEs are poorly understood. Dang et al. characterized the AE group member (MoAE4) in *Magnaporthe oryzae*. MoAE4 exhibits more sequence and structure homologies with the reported AE4 and BOR1 proteins [8]. Summarily, the data delineate a cytomembrane- and tonoplast-located HCO_3_^−^ transporter, which is required for development and pathogenicity in *M. oryzae*, revealing a potential drug target for blast disease control.

MicroRNA plays an important role in multifarious biological processes by regulating their corresponding target genes. However, the biological function and regulatory mechanism of fungal microRNA-like RNAs (milRNAs) remain poorly understood. In a study [9], combined with deep sequencing and bioinformatics analysis, milRNAs and their targets from *Trichoderma guizhouence* NJAU 4742 were isolated and identified under solid-state fermentation (SSF) using rice straw as the sole carbon source. The authors concluded that Tr-milRNA1 from NJAU 4742 improved lignocellulose utilization under heat stress by regulating the expression of the corresponding target gene Trvip36. These findings might open avenues for exploring the mechanism of lignocellulase secretion in filamentous fungi.

Light is perceived by photoreceptors in fungi and further integrated into the stress-activated MAPK HOG pathway, and thereby potentially activates the expression of genes for stress responses. The precise control of light conditions can likely improve the conidial yield and stress resistance to guarantee the low cost and long shelf life of Trichoderma-based biocontrol agents and biofertilizers. In a study [10], effects of wavelengths and intensities of light on conidial yield and stress tolerance to osmotic, oxidative and pH stresses in *Trichoderma guizhouense* were investigated. The authors found that blue light increased the conidial yield more than 1000 folds as compared to dark conditions and simultaneously enhanced conidial stress resistance.

Enzymatic catalysis is one of the main pillars of sustainability for industrial production. Enzyme application allows the use of toxic solvents to be minimized and the valorization of agro-industrial residues through reuse [11]. In addition, the enzymes are safe and energy-efficient. Nonetheless, their use in biotechnological processes is still hindered by their cost, stability and low rate of recycling and reuse. Among the many industrial enzymes, fungal laccases (LCs) are perfect candidates to serve as a biotechnological tool, as they are outstanding, versatile catalytic oxidants, only requiring molecular oxygen to function. Loi et al. [11] presented an overview of fungal LC (a promising green and sustainable enzyme), its mechanism of action, advantages, disadvantages, and solutions for its use as a tool to reduce the environmental and economic impact of industrial processes with a particular insight on the reuse of agro-wastes.

As a fundamental mineral element for cell growth and development, iron is available for uptake as ferric ions, which are usually oxidized into complex oxyhydroxide polymers, insoluble under aerobic conditions. In these conditions, the bioavailability of iron is dramatically reduced. As a result, microorganisms face problems of iron acquisition, especially under low concentrations of this element. However, some microbes have evolved mechanisms for obtaining ferric irons from the extracellular medium or environment by forming small molecules often regarded as siderophores. Pecoraro et al. systematically reviewed biosynthesis pathways, transport mechanisms and biotechnological applications of fungal siderophores [12].

With the increasing world population, demand for industrialization has also increased to meet humans’ living standards. Fungi are considered a source of essential constituents to produce biocatalytic enzymes, including amylases, proteases, lipases, and cellulases, with broad-spectrum industrial and emerging applications. Naeem et al. discussed the origin, nature, mechanism of action, emerging aspects of genetic engineering for designing novel proteases, genome editing of fungal strains through CRISPR technology, present challenges and future recommendations of fungal proteases [13]. The emerging evidence revealed that CRISPR/Cas9 technology had been successfully developed in various filamentous fungi and higher fungi for the editing of specific genes. In addition to medical importance, fungal proteases are extensively used in different industries such as foods to prepare butter, fruits, juices and cheese, and to increase their shelf life. It is concluded that hydrolysis of proteins in industries is one of the most significant applications of fungal enzymes that led to massive usage of proteomics.

Alternaria is a ubiquitous fungal genus in many ecosystems, consisting of species and strains that can be saprophytic, endophytic or pathogenic to plants or animals, including humans. Alternaria species can produce a variety of secondary metabolites (SMs), especially low-molecular-weight toxins. Wang et al. [14] reviewed the recent advances in Alternaria phytotoxins, which covered the classification, chemical structure, occurrence, bioactivity and biosynthesis of the major Alternaria phytotoxins.

Overall, this Special Issue provides up-to-date findings related to fungal biotechnologies and their application in bioenergy production, agro-industrial waste utilization, etc. We would like to take this opportunity to thank Prof. David S. Perlin, the Editor-in-Chief of the *Journal of Fungi*, for giving us this great opportunity to publish a Special Issue “Fungal Biotechnology and Applications”. We also appreciate the contributions from microbiological experts and congratulate them for their significant findings and hard work. We also owe our peer reviewers a huge debt of gratitude for their insightful comments that helped to improve the quality of manuscripts published in this issue.
